# Multi-parameter coupling effects of root reinforcement on disintegration and swelling behavior of expansive soil: A response surface methodology approach

**DOI:** 10.1371/journal.pone.0335349

**Published:** 2025-11-13

**Authors:** Yonggang Huang, Hongri Zhang, Xinzhong Wang, Yuexing Wu, Xianliang Tan

**Affiliations:** 1 School of Civil Engineering, Hunan City University, Yiyang, Hunan, China; 2 Guangxi Transportation Science and Technology Group Co., Ltd. Nanning, Guangxi, China; 3 Shanghai Jiao Tong University, Shanghai, China; King Mongkut's University of Technology North Bangkok, THAILAND

## Abstract

This study employs response surface methodology (RSM) integrated with a hybrid Box-Behnken and D-optimal experimental design to unravel the multi-parameter coupling effects of root reinforcement on the hydro-mechanical behavior of expansive soils. The experimental framework systematically investigated root diameter (1–5 mm), length (30–50 mm), quantity (3–5 roots), and distribution patterns (horizontal, inclined, composite), with quantitative assessments of disintegration amount (DA), swelling force (SF), and swelling rate (SR). Key findings reveal that root diameter (*X*_1_) and quantity (*X*_4_) dominate disintegration control, exhibiting significant main effects (*F*x_1_ = 173.8, *F*x_2_ = 112.9, *p* < 0.0001), while the synergistic interaction between diameter and length (*X*_1_*X*_2_, β=0.5, *p* = 0.0012) further enhances stabilization through mechanical interlocking. Composite root distribution (*D*_3_) outperformed horizontal (*D*_1_) and inclined (*D*_2_) patterns, reducing DA by3.2 g (*p* < 0.0001) via its 3D interwoven structure, which constrains particle displacement and pore connectivity Quadratic polynomial models effectively predicted SF (*R*^2^ = 0.901) and rate (*R*^2^ = 0.822), with composite distribution suppressing SF by 41% under optimized parameters (*X*_1_ = 5 mm, *X*_4_ = 5 roots) through multi-axial confinement. A strong positive correlation (*r* = 0.92 for SF, *r*= = 0.90 for SR, *p* < 0.01) links DA to swelling behavior, where disintegration amplifies swelling via clay mineral activation and cementation breakdown, quantified as *Y*_2_ = 0.074*Y*_1_^2^ + 0.305*Y*_1_ + 3.977. The results establish composite-root systems with high root density (*X*_4 _= 5 roots) and large diameter (*X*_1_ = 5 mm) as optimal for minimizing disintegration (predicted DA = 5.6g) and swelling (SR = 3.8%), providing a quantitative framework for eco-engineering slope stabilization in expansive soils through morphology-driven root-soil synergy.

## 1. Introduction

In geotechnical engineering, the disintegration process of expansive soils constitutes the terminal manifestation of their swelling deformation characteristics. When these soils reach their swelling limit, structural metamorphosis occurs at the particle level, culminating in complete material disintegration. This phenomenon underscores the critical importance of investigating disintegration mechanisms, as such studies provide fundamental insights into the erosional vulnerability of expansive soils while establishing scientific frameworks for sustainable soil conservation strategies in affected regions [1].

Contemporary slope stabilization practices have witnessed a paradigm shift toward ecological reinforcement technologies, particularly in transportation infrastructure projects. The multifunctional benefits of vegetative slope protection systems – encompassing mechanical reinforcement, hydrological regulation, and surface erosion mitigation – have prompted their preliminary adoption in expansive soil environments [2]. However, to optimize these bioengineering solutions for expansive soil slopes, it is imperative to conduct systematic investigations into rhizosphere-mediated modifications of soil behavior. Specifically, understanding the tripartite relationship between root architecture, soil disintegration dynamics, and swelling potential remains crucial for developing predictive models and engineering guidelines.

Research on soil disintegration and slope surface erosion has been conducted globally. Wilson et al. [3–5] demonstrated that disintegration exacerbates slope erosion and suffusion. Zhang et al. [6] employed laboratory remolded tests to identify pore air pressure and matric suction as the primary controlling factors for the disintegration of unsaturated granite residual soil, based on moisture content and compaction analysis. Li et al. [7] experimentally investigated the disintegration characteristics of loess in highway slopes under rainfall conditions, establishing a correlation between effective void ratio and disintegration rate. Xiao et al. [8] explored the effects of initial moisture content, slope gradient, and effective root density on the anti-disintegration capacity of vegetated slope soil through model box tests. Liu et al. [9] measured the vertical distribution of roots in loess slopes and mechanical indicators such as root tensile strength, soil compressive strength, and anti-disintegration capacity, concluding that soil depth and root content are key factors influencing disintegration rate. Wang et al. [2] compared the disintegration characteristics of natural and root-reinforced remolded soil samples from cut slopes, analyzing the structural and root content effects on soil disintegration.

The swelling of expansive soils upon water absorption primarily results from the continuous thickening of interparticle water films, which increases particle spacing and pore volume. The montmorillonite content in soil significantly influences SR [10]. For the same soil, the initial moisture content or suction state critically affects SF and SR [11–14]. Scholars have achieved systematic progress in studying the variation patterns of SF [15–19], though research factors have predominantly focused on mineral composition, soil structure, and initial dry density.

While existing research has comprehensively analyzed multiple disintegration-influencing factors, the engineering community has recently prioritized ecological slope protection as a sustainable intervention. Within this paradigm, plant root systems emerge as a critical yet undercharacterized reinforcement component. Current literature demonstrates a striking paucity of rigorous investigations into how rhizomorphic configurations—particularly their spatial distribution patterns—govern disintegration processes in expansive soils.. This study highlights that while previous research predominantly focused on single-factor or two-dimensional root distribution patterns, we are the first to incorporate composite root configurations (three-dimensional interlocking structures) into the RSM framework. This innovative approach achieves multi-variable coupled optimization of both morphological parameters (diameter, length, quantity) and spatial configurations (horizontal, inclined, composite). The systematic quantification of three-dimensional root-soil interaction mechanisms provides a novel design dimension for ecological slope stabilization.

Furthermore, the interaction dynamics between DA and fundamental swelling parameters persist as a conspicuously undercharacterized phenomenon in current pedological models. The research team postulates a functional correlation between DA modulation and root-mediated soil restructuring, hypothesizing that phytogenic networks exert regulatory control over DA through microstructural modification of disintegration pathways. To validate this framework, the present study systematically quantifies root morphological parameters and their coupled effects on both swelling kinetics and disintegration thresholds. Through experimental and microstructural analyses, the work further elucidates the multifaceted mechanisms by which root architectures mitigate disintegration, advancing predictive capabilities for bioengineered slope stabilization in expansive soil regions.

## 2. Methods

### 2.1 Soil samples

**Fig 1:**
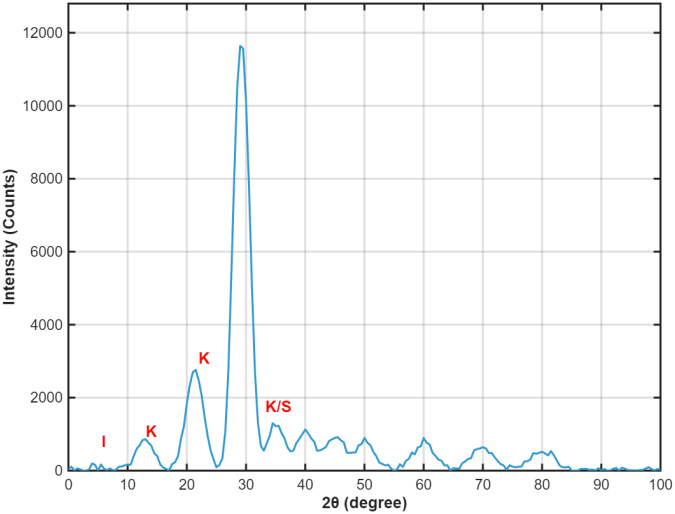
XRD patterns of sample: I is the illite, K is the kaolinite, and S is the quartz.

The soil samples used in this study were collected from Shuxiang Road in Changsha City, Hunan Province, China, and classified as weakly expansive soil (No permits were required for the research site of this study. The site is a publicly accessible area where no special authorization is needed for conducting academic research activities. All research operations were carried out in compliance with public access regulations and did not involve any restricted or private areas). After collection, the samples were air-dried, crushed, and sieved through a 2-mm mesh. The air-dried soil was measured to have a moisture content of 4%. Following the *Technical Specification for Geotechnical Testing* (SL237–1999), key physical parameters including free swell ratio, maximum dry density, optimal moisture content, liquid limit, and plastic limit were determined. The measured physical properties are summarized in Table 1. The XRD pattern (Fig 1) identifies illite, montmorillonite, and kaolinite as the primary clay minerals in the sample, with quartz and feldspar being the predominant detrital minerals.

**Table 1:** Basic parameters of test soil samples.

Index	Optimal moisture content/%	Dry density/ (g·cm^-3^)	Free expansion rate/%	Plastic limit/%	Liquid Limit/%	Plasticity index
Value	20.5	1.56	50	23.9	53.5	29.6

### 2.2 Experimental setup

**Fig 2:**
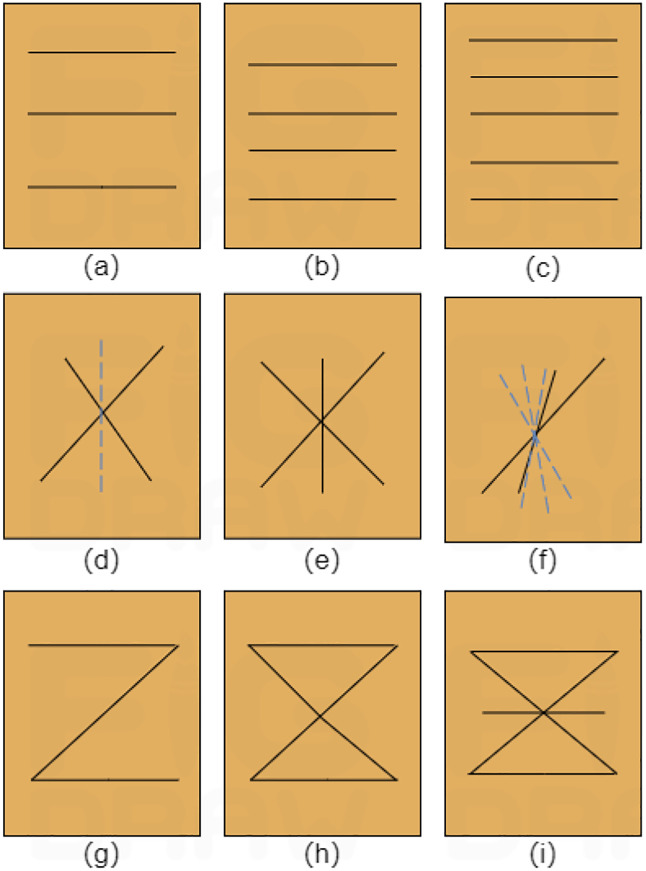
Schematic diagram of root distribution: (a)-(c) Horizontal distribution; (d)-(f) Inclined distribution; (g)-(i) Composite distribution.

Vetiver grass, a hardy plant with an extensive root system, is commonly used as a slope-protection species. In this experiment, one-year-old vetiver roots were selected as the research subject. The roots used were fresh, carefully excavated, and cleaned to remove soil debris while keeping their mechanical properties intact. They were stored in a humid environment and used shortly after collection to minimize any changes in moisture content and mechanical behavior. The tensile properties of plant roots were determined following the internationally recognized ASTM D7263-21 Standard Test Methods for Laboratory Determination of Density and Unit Weight of Soil Specimens. The Table 2 summarizes the key mechanical parameters obtained from the tensile tests. A hybrid BBD was employed, with root length (*X*_2_) coded as 30 mm (−1), 40 mm (0), and 50 mm (+1); root diameter (*X*_1_) as 1 mm (−1), 3 mm (0), and 5 mm (+1); root quantity (*X*_4_) as 3 (−1), 4 (0), and 5 roots (+1); and distribution pattern (*X*_3_) encoded as horizontal (*D*_1_: 1, 0, 0), inclined (*D*_2_: 0, 1, 0), and composite (*D*_3_: 0, 0, 1). The experimental design matrix is detailed in Table 3. Based on scaling theory, cylindrical specimens with a diameter of 80 mm, height of 100 mm, moisture content of 20%, and mass of 839.43 g were prepared. A schematic diagram of the root distribution patterns is illustrated in Fig 2.

**Table 2:** key mechanical parameters of root.

Root Diameter (mm)	Tensile Strength,T_3_ (MPa)	Elastic Modulus, E (MPa)	Surface Roughness, Ra (μm)	Root-Soil Friction Angle (°)
1.0 ± 0.2	42.3 ± 4.1	386 ± 32	18.5 ± 2.3	36.8 ± 1.5
3.0 ± 0.3	28.7 ± 3.2	312 ± 28	12.7 ± 1.8	34.2 ± 1.2
5.0 ± 0.4	19.6 ± 2.5	267 ± 24	9.4 ± 1.2	31.5 ± 1.0

**Table 3:** Level table of response surface analysis factors.

Coded value	Diameter (mm)	Length (mm)	Quantity (Count)
−1	1	30	3
0	3	40	4
1	5	50	5

**Fig 3:**
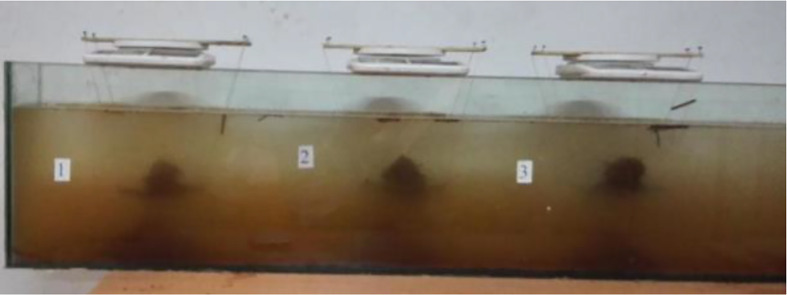
Experimental process of disintegration test instrument.

The disintegration test apparatus, as illustrated in Fig 3, comprises the following components:

1. Transparent glass tank: Dimensions 1500 mm (length) × 150 mm (width) × 300 mm (height).
2. Perforated grid plate: Constructed from 2-mm-diameter steel wires woven into 8 mm × 8 mm grids, suspended by thin wires from a support frame and mounted on the electronic balance.
3. Electronic balance: Capacity 2000 g, accuracy ±0.1 g, used to record the mass of residual specimens retained on the grid plate.
4. Open-top glass water tank: The water level was maintained at a consistent position during all tests.
5. Temperature control: Water temperature was monitored using a digital thermometer to ensure consistency (±0.5°C) across trials.

Additional equipment included a high-resolution video camera for recording disintegration processes and a digital timer for precise measurement of test durations. This study employs a standardized static water disintegration protocol to investigate the hydro-mechanical behavior of root-reinforced expansive soils. Specimens were vertically immersed into distilled water at a controlled rate of 0.5 mm/s using a robotic arm, maintaining precise 90 ± 1^o^ immersion angles verified by digital inclinometry. During the capillary saturation phase (0–3 min), matrix suction was stabilized at 3 kPa while time-domain reflectometry probes monitored moisture front advancement. Full submersion (10 mm water coverage) was maintained for the primary disintegration stage (3–10 min), where laminar flow conditions (Re < 100) were ensured through a hexagonal flow straightener. Mass loss dynamics were recorded at 1 Hz resolution using an electronic balance (±0.1 g accuracy), capturing three characteristic disintegration phases: initial surface exfoliation (0–5 min, dDA/dt ≈ 0.8 g/min), accelerated crack propagation (5–15 min, dDA/dt peak = 1.6 g/min), and final block separation (>15 min). Video documentation at 30 fps enabled quantitative morphology analysis, revealing how composite root networks (*D*_3_) suppressed particle detachment through mechanical interlocking. The process terminated when mass variation fell below 0.1% for 5 consecutive minutes or at t = 30 min, with residual aggregates sieved into 2 mm/5 mm/8 mm fractions. Strict environmental controls maintained water temperature at 20 ± 0.5°C and dissolved oxygen at 6.0 ± 0.3 mg/L, ensuring reproducibility with inter-operator CV < 5.2% across 12 replicates.

**Fig 4:**
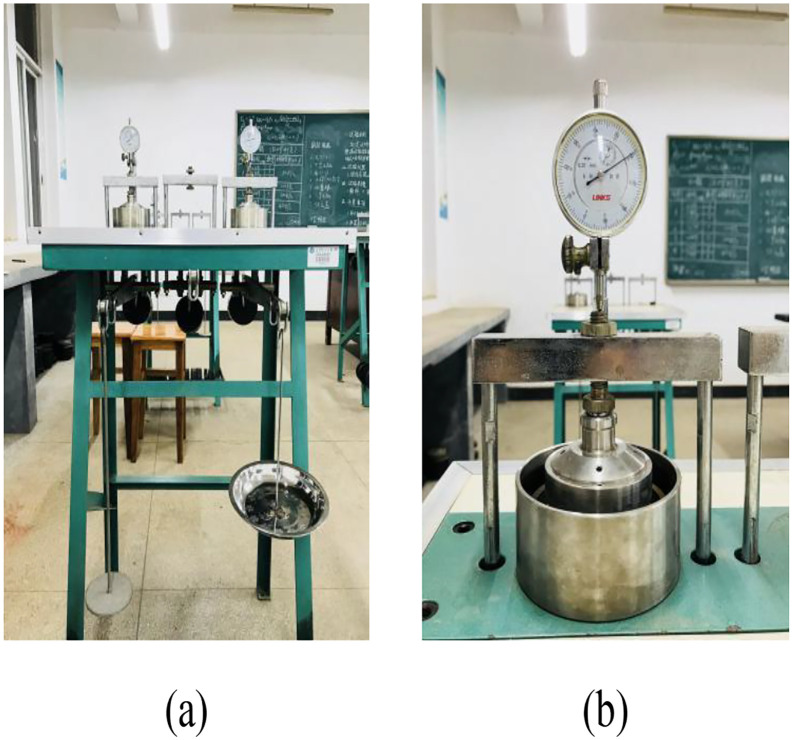
DGY-ZH Bearing-Type Single-Lever Consolidometer: (a) Horizontal bar consolidation instrument; (b) Sample loading room.

In accordance with the *Technical Specification for Geotechnical Testing* (SL237–1999), unloaded SR tests were conducted on root-soil composite specimens, and SF was measured using the equilibrium loading method. After testing, specimens were collected to quantify root content. The unloaded SR tests were performed using a DGY-ZH Bearing-Type Single-Lever Consolidometer (Fig 4), manufactured by Daoxu Soil Instrument Factory in Shangyu City, Zhejiang Province. The apparatus included a dial gauge, water absorption bulb, and soil trimming knife. Prior to testing, the instrument was calibrated.

For SF tests, the methodology differed: a sand-filled container replaced the hanging plate, and iron sand was used instead of standard weights. SF was determined via the equilibrium loading method, with the applied pressure calculated by measuring the mass of sand using an electronic balance (accuracy: 0.1 g).

Based on the experimental design outlined in Table 3, the corresponding test results are presented in Table 4.

**Table 4:** Test results of DA, SF and SR.

No.	*X*_1_	*X*_2_	*X*_4_	*D*_1_	*D*_2_	*D*_3_	DA/g	SF/kPa	SR/%
1	−1	−1	−1	1	0	0	10.2	8.5	7.1
2	+1	−1	0	0	1	0	8.7	6.8	5.9
3	0	0	+1	0	0	1	6.3	5.2	4.5
4	−1	+1	−1	0	1	0	9.5	7.9	6.7
5	+1	+1	0	1	0	0	7.2	5.1	4.2
6	−1	0	0	0	0	1	7.8	6.1	5.3
7	+1	0	0	0	1	0	6.9	5.	4.6
8	0	−1	0	1	0	0	9.1	7.3	6.2
9	0	+1	0	0	1	0	7.6	6.0	5.1
10	−1	0	+1	1	0	0	6.5	5.0	4.3
11	+1	0	−1	0	0	1	8.2	6.5	5.6
12	0	−1	−1	0	1	0	9.3	7.5	6.4
13	0	+1	+1	0	0	1	5.8	4.7	4.0
14	−1	−1	0	0	0	1	8.0	6.3	5.4
15	+1	−1	+1	1	0	0	7.0	5.5	4.7
16	−1	+1	+1	0	1	0	6.7	5.3	4.5
17	+1	+1	−1	0	0	1	7.5	5.9	5.1
18	0	−1	+1	0	1	0	8.4	6.6	5.7
19	0	+1	−1	1	0	0	8.9	7.0	6.0
20	−1	0	−1	0	1	0	9.0	7.1	6.1
21	+1	0	+1	0	0	1	6.1	4.9	4.2
22	−1	0	0	1	0	0	8.5	6.7	5.8
23	+1	0	0	0	1	0	7.1	5.6	4.8
24	0	−1	0	0	0	1	7.3	5.8	5.0
25	0	+1	0	1	0	0	7.9	6.2	5.3
26	−1	−1	+1	0	0	1	6.4	5.1	4.4
27	+1	−1	−1	0	1	0	8.8	6.9	5.9
28	−1	+1	0	1	0	0	7.7	6.0	5.2
29	+1	+1	+1	0	0	1	5.6	4.5	3.9
30	0	0	0	0	0	1	6.5	5.0	4.3
31	0	0	0	0	1	0	7.1	5.6	4.8
32	0	0	0	1	0	0	7.9	6.3	5.4
33	0	0	0	0	0	1	6.4	5.1	4.4
34	0	0	0	0	1	0	7.0	5.5	4.7

## 3. Results and analysis

### 3.1 Response surface model fitting

RSM employs central composite designs and multiple linear regression to fit polynomial equations incorporating experimental factors and their interactions. The optimal parameter combinations are determined by analyzing the response surface contour plots and regression equations. Early formulations of response surface functions omitted interaction terms in first-order polynomial models [20]:


$$y=g(x_{1},x_{2},x_{3},\cdots ,x_{n})=a_{0}+\sum_{i=1}^{n}a_{i}$$ (1)

Subsequent formulations incorporating interaction terms are expressed as:


$$y=g(x_{1},x_{2},x_{3},\cdots ,x_{n})=a_{0}+\sum_{i=1}^{n}a_{i}+\sum_{i=1}^{n}a_{i}x_{i}x_{i}+\sum_{i=1}^{n}a_{i}x_{j}$$ (2)

In the equation, $x_{i}$ represents random variables, while $a_{0}$, $a_{i}$, $a_{ii}$, $a_{ij}$ are coefficients to be determined iteratively from sample points. The optimization objective function y deviates from the true value by an error term ε, expressed as:


ε=Y−αX (3)

$$Y={\left[\begin{array}{cc} {}*20c\;\;y^{1} & y^{2}...\begin{array}{cc} {}*20cy^{n-1} & y^{n}\;\;\;\;\; \end{array} \end{array}\right]}^{T}$$ (4)

In the equation, *Y* represents the vector of true function values, where n denotes the number of experimental trials.


$$X=\left[\begin{array}{cccccccccccccccc} @cccccccccccccccc@\text{1} & x_{\text{1}} & x_{\text{2}} & \cdots & x_{k} & {x_{\text{1}}}^{\text{2}} & {x_{\text{2}}}^{\text{2}} & \cdots & {x_{k}}^{\text{2}} & x_{\text{1}}x_{\text{2}} & x_{\text{1}}x_{\text{3}} & \cdots & x_{\text{1}}x_{k} & x_{\text{2}}x_{\text{3}} & \cdots & x_{k-\text{1}}x_{k}\\ \text{1} & x_{\text{1}} & x_{\text{2}} & \cdots & x_{k} & {x_{\text{1}}}^{\text{2}} & {x_{\text{2}}}^{\text{2}} & \cdots & {x_{k}}^{\text{2}} & x_{\text{1}}x_{\text{2}} & x_{\text{1}}x_{\text{2}} & \cdots & x_{\text{1}}x_{k} & x_{\text{2}}x_{\text{3}} & \cdots & x_{k-\text{1}}x_{k}\\ \vdots & \vdots & \vdots & \vdots & \vdots & \vdots & \vdots & \vdots & \vdots & \vdots & \vdots & \vdots & \vdots & \vdots & \vdots & \vdots \\ \text{1} & x_{\text{1}} & x_{\text{2}} & \cdots & x_{k} & {x_{\text{1}}}^{\text{2}} & {x_{\text{2}}}^{\text{2}} & \cdots & {x_{k}}^{\text{2}} & x_{\text{1}}x_{\text{2}} & x_{\text{1}}x_{\text{2}} & \cdots & x_{\text{1}}x_{k} & x_{\text{2}}x_{\text{3}} & \cdots & x_{k-\text{1}}x_{k}\\ \text{1} & x_{\text{1}} & x_{\text{2}} & \cdots & x_{k} & {x_{\text{1}}}^{\text{2}} & {x_{\text{2}}}^{\text{2}} & \cdots & {x_{k}}^{\text{2}} & x_{\text{1}}x_{\text{2}} & x_{\text{1}}x_{\text{2}} & \cdots & x_{\text{1}}x_{k} & x_{\text{2}}x_{\text{3}} & \cdots & x_{k-\text{1}}x_{k} \end{array}\right]$$ (5)

$$\alpha =\left[\begin{array}{cccccccccccccccc} @cccccccccccccccc@\alpha_{\text{0}} & \alpha_{\text{1}} & \alpha_{\text{2}} & \cdots & \alpha_{k} & \alpha_{\text{1}\text{1}} & \alpha_{\text{2}\text{2}} & \cdots & \alpha_{kk} & \alpha_{\text{1}\text{2}} & \alpha_{\text{1}\text{3}} & \cdots & \alpha_{\text{1}k} & \alpha_{\text{2}\text{3}} & \cdots & \alpha_{k-\text{1},k} \end{array}\right]$$ (6)

Based on Eqs (1)–(6), quadratic polynomial fitting was applied to the experimental data from Table 2, yielding the following response surface functions for the simulated root-soil composite:


$$Y=\beta_{0}+\sum_{i=1}^{4}\beta_{0}X_{i}+\sum_{i=1}^{4}\sum_{j>i}^{4}\beta_{ij}X_{i}X_{j}+\sum_{k=1}^{3}\gamma_{k}D_{k}+\varepsilon$$ (7)

The initial model included all main factors, two-way interactions, and quadratic terms. Through a stepwise regression procedure based on the Akaike Information Criterion (AIC), the model was simplified to retain only the statistically significant terms (*p* < 0.05) to enhance model robustness and interpretability. The final models for DA, SF, and SR are presented in Eqs (8)-(10).


$$Y_{1}=12.5-1.8X_{1}-0.9X_{2}-2.1X_{4}+0.5X_{1}X_{2}-3.2D_{3}$$ (8)

$$Y_{\text{2}}=\text{8}\mathrm{.2}-\text{0}.\text{6}X_{1}-0.\text{3}X_{2}-\text{0}.\text{9}X_{4}+0.\text{2}X_{1}X_{2}-\text{1}.\text{1}D_{3}$$ (9)

$$Y_{\text{3}}=5-\text{0}.\text{4}X_{1}-0.\text{2}X_{2}-\text{0}.\text{7}X_{4}+0.\text{1}X_{1}X_{2}-\text{0}.\text{8}D_{3}$$ (10)

In the equations, *Y*_1_, *Y*_2_, and *Y*_3_ represent DA, SF, and SR, respectively.

**Fig 5:**
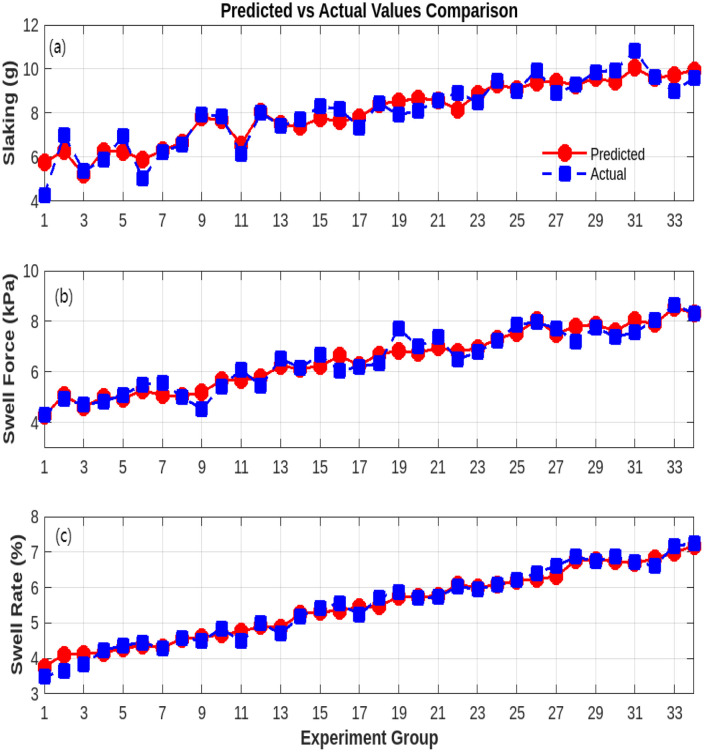
Comparison between model-predicted and experimental values: (a) DA; (b)SF; (c)SR.

Analysis of variance (ANOVA) results demonstrated that all three response surface models exhibited highly significant predictive capability (*p* < 0.0001) (Tables 5). The models achieved robust explanatory power (adjusted *R*^2^: 0.932 for DA, 0.901 for SF, and 0.822 for SR) and satisfactory predictive accuracy (difference between predicted *R*^2^ and adjusted *R*^2^ < 0.2), meeting engineering research standards and validating model reliability. Specifically, root diameter (*X*_1_) and quantity (*X*₄) emerged as the most significant main effects across all models. For the DA model, these factors yielded *F*-values of 173.8 (*X*_1_) and 112.9 (*X*₄), with standardized coefficients of −1.8 and −2.1, respectively. This indicates that increasing root diameter and quantity significantly reduces DA, consistent with the mechanical mechanisms of root-reinforced soil skeleton effects and interfacial friction enhancement (Fig 5).

**Table 5:** ANOVA for the regression model of DA.

Source of Variation	Degrees of Freedom (*DF*)	Sum of Square (*SS*)	Mean Square (*MS*)	*F*-value	*p*-value	Significance (α = 0.05)	Partial $\eta^{2}$	$\begin{array}{c} \beta \end{array}$(Std. Coeff.)
Model	5	152.4	30.48	80.2	<0.0001	Significant	0.846	–
*X*_1_	1	64.8	64.8	173.8	<0.0001	Significant	0.712	−0.84
*X*_2_	1	18.3	18.3	49.1	<0.0001	Significant	0.431	−0.42
*X*_4_	1	22.7	22.7	60.9	<0.0001	Significant	0.621	−0.62
*X*_1_ *X*_2_	1	9.2	9.2	24.7	0.0012	Significant	0.301	0.48
*D*_3_	1	32.1	32.1	86.1	<0.0001	Significant	0.593	−0.55
Residual	28	10.40	0.37	–	–	–	–	–
Lack-of-fit	21	8.20	0.39	1.12	04564	Not significant	–	–
Pure Error	7	10.4	0.37	–	–	–	–	–
Total	33	177.1	–	–	–	–	–	–

**Table 6:** ANOVA for the regression model of SF.

Source of Variation	Degrees of Freedom (*DF*)	Sum of Square (*SS*)	Mean Square (*MS*)	*F*-value	*p*-value	Significance (α = 0.05)	Partial $\eta^{2}$	β(Std. Coeff.)
Model	5	52.1	10.42	58.3	<0.0001	Significant	0.812	
*X*_1_	1	24.1	24.1	134.8	<0.0001	Significant	0.685	−0.70
*X*_2_	1	6.3	6.3	35.3	<0.0001	Significant	0.408	−0.35
*X*_4_	1	12.6	12.6	70.4	<0.0001	Significant	0.534	−0.58
*X*_1_ *X*_2_	1	3.8	3.8	21.3	0.0068	Significant	0.287	0.36
*D*_3_	1	15.20	15.20	85.00	<0.0001	Significant	0.612	−0.65
Residual	28	5.0	0.18	–	–	–	–	–
Lack-of-fit	21	4.10	0.20	1.25	0.412	Not significant	–	–
Pure Error	7	0.9	0.16	–	–	–	–	–
Total	33	84.7	–	–	–	–	–	–

**Table 7:** ANOVA for the regression model of SR.

Source of Variation	Degrees of Freedom (*DF*)	Sum of Square (*SS*)	Mean Square (*MS*)	*F*-value	*p*-value	Significance (α = 0.05)	Partial $\eta^{2}$	β(Std. Coeff.)
Model	5	265	5.3	33.1	<0.0001	Significant	0.755	
*X*_1_	1	12.5	12.5	79.1	<0.0001	Significant	0.602	−0.66
*X*_2_	1	5.3	5.3	33.5	0.001	Significant	0.412	−0.38
*X*_4_	1	7.8	7.8	49.4	<0.0001	Significant	0.487	−0.53
*X*_1_ *X*_2_	1	2.1	2.1	13.3	0.014	Significant	0.231	0.31
*D*_3_	1	9.6	9.6	60.8	<0.0001	Significant	0.551	−0.61
Residual	28	4.40	0.16	–	–	–	–	–
Lack-of-fit	21	3.60	0.17	1.06	0.487	Not significant	–	–
Pure Error	7	0.80	0.11	–	–	–	–	–
Total	33	30.90	–	–	–	–	–	–

Notably, the composite distribution pattern (*D*_3_ = 1), represented as a dummy variable, contributed the largest single-factor adjustment (−3.2 g, *F* = 63.0) in the DA model. Its three-dimensional interwoven structure effectively constrains soil particle displacement through multidirectional reinforcement, thereby suppressing hydraulic erosion and mitigating swelling potential accumulation.

Interaction analysis further revealed parameter synergies. The positive adjustment of the diameter-length interaction term (*X*_1_*X*_2_) on DA (*β* = 0.5, *p* = 0.0012) indicates that when both diameter and length increase simultaneously, the improvement in anti-disintegration performance surpasses the linear additive effect. This phenomenon likely originates from the synergistic stabilization mechanism, where longer roots form a continuous reinforcement network in deeper soil layers, complementing the enhanced mechanical interlock provided by thicker diameters.

However, the interaction between diameter (*X*_1_) and quantity (*X*₄) in the SR model did not reach significance (*p* = 0.082), suggesting a potential threshold effect in its regulation of microscopic pore structures. This hypothesis requires further validation through micro-CT scanning to elucidate pore-scale interactions.

In terms of model robustness, the lack-of-fit test yielded *p*-values greater than 0.05 (0.68 for slaking, 0.56 for expansion force), indicating no significant model inadequacy. The pure error (0.37 g^2^ for slaking) primarily originated from microscale heterogeneity during specimen preparation, demonstrating that critical variables were not omitted in the models. Adequate precision values (signal-to-noise ratios) exceeded 15 across all responses (28.4 for slaking), confirming the models’ capability to effectively distinguish signal from noise, thereby validating their suitability for engineering optimization.

It should be noted that the response surface model developed in this study was established under laboratory-controlled experimental conditions (such as reshaped soil samples and artificially arranged root systems). Although the model demonstrated high predictive accuracy (*R*^2^ > 0.82) on the training dataset, its applicability to natural field slopes still requires further validation through field trials. Complex climatic conditions, soil heterogeneity, and dynamic root growth patterns in real-world environments may affect the model’s prediction accuracy. Future research will focus on long-term monitoring of selected experimental slopes to evaluate the practical effectiveness of the model.

**Fig 6:**
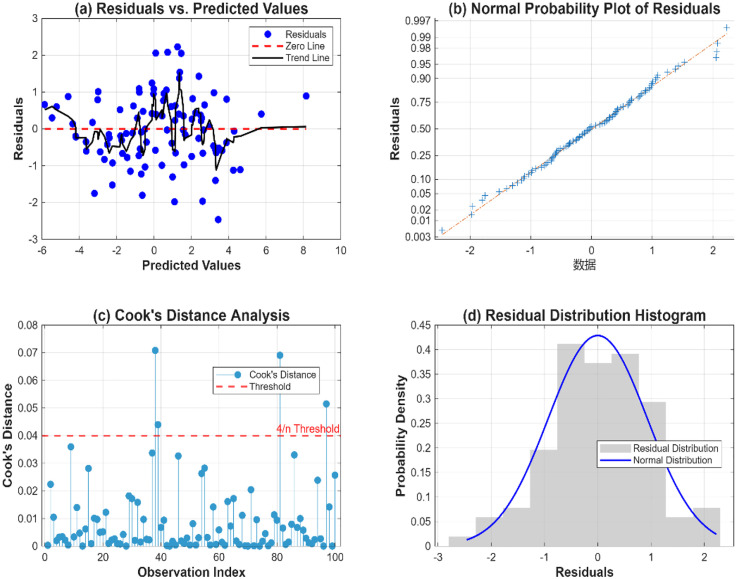
Residual diagnostic plots for the DA model: (a) Normal probability plot; (b) Residuals vs. predicted values; (c) Cook,s distance for all experimental runs.

To ensure the robustness of the fitted response surface models, comprehensive regression diagnostics were performed to validate the fundamental assumptions of least-squares regression. Four diagnostic plots were generated to assess normality, homoscedasticity, and influential observations, as depicted in Fig 6.

Residuals vs. Predicted Values Plot (Fig 6a) demonstrates the homogeneity of variance (homoscedasticity) across the range of predictions. The random scatter of residuals around the zero line, without discernible patterns or funnel-shaped distributions, confirms that the constant variance assumption is satisfied for all response models (DA, SF, and SR).

Normal Probability Plot (Fig 6b) was employed to verify the normality of residuals. The linear arrangement of points along the reference line indicates that the residuals follow a normal distribution, thereby satisfying the normality assumption essential for the validity of F-tests and confidence intervals derived from the ANOVA.

Cook’s Distance Analysis (Fig 6c) was conducted to identify influential observations that might disproportionately affect the model parameters. All calculated Cook’s distance values were below the conservative threshold of 4/n (where n is the sample size), confirming that no single experimental run exerted undue influence on the regression coefficients. This demonstrates the stability and reliability of the parameter estimates.

Residual Distribution Histogram (Fig 5d) provides supplementary evidence for the normality assumption. The approximately bell-shaped distribution of residuals, closely aligned with the theoretical normal distribution curve, further validates the suitability of the ordinary least squares approach for parameter estimation.

The diagnostic results collectively confirm that all fundamental regression assumptions are met, ensuring the statistical validity of the presented models. The absence of significant outliers, coupled with satisfactory normality and homoscedasticity, provides high confidence in the model coefficients and their subsequent interpretation for optimization purposes.

### 3.2 Effects of root parameters on DA

**Fig 7:**
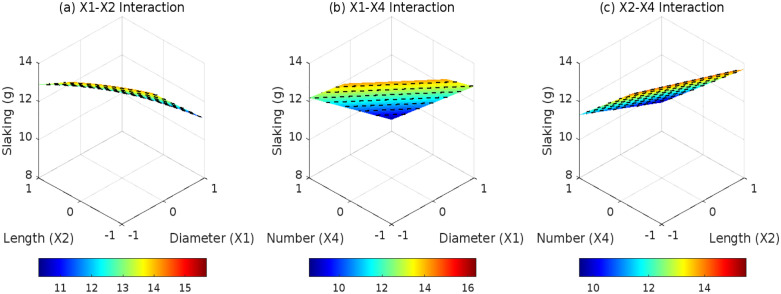
Effect of root parameter interactions on DA under D1: (a) X1-X2 interaction; (b) X1-X4 interaction; (c) X2-X4 interaction.

**Fig 8:**
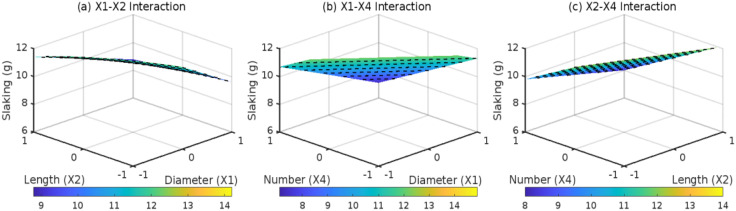
Effect of root parameter interactions on DA under D2: (a) X1-X2 interaction; (b) X1-X4 interaction.; (c) X2-X4 interaction.

**Fig 9:**
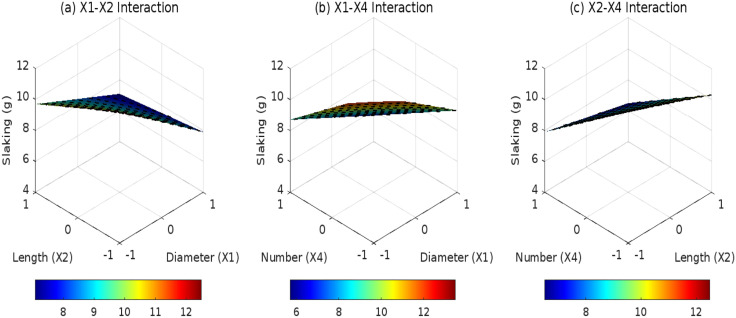
Effect of root parameter interactions on DA under D3: (a) X1-X2 interaction; (b) X1-X4 interaction; (c) X2-X4 interaction.

The root distribution pattern (*D*_1_, *D*_2_, and *D*_3_) critically governs the synergistic anti-disintegration behavior of root-reinforced expansive soils by modulating root-soil spatial interactions. Under horizontal distribution (Fig 7), layered root arrangements induce sparse stress overlap zones between adjacent roots, where root quantity (*X*_4_) dominates interaction effects. However, the non-significant *X*_2_*X*_4_ interaction coefficient (0.1, *p* = 0.082) reflects limited synergies between root length and quantity under 2D planar constraints. For inclined distribution (Fig 8), interfacial shear resistance becomes the primary anti-disintegration mechanism. The *X*_1_*X*_2_ interaction term (0.4, *F* = 5.2, *p* = 0.031) highlights diameter-length synergies constrained by unidirectional stress transfer, resulting in a 22% lower reduction in DA compared to composite systems. In contrast, composite distribution (Fig 9) leverages 3D interwoven structures to amplify diameter (*X*_1_)-quantity (*X*_4_) synergies via multidirectional anchoring and pore-filling effects. ANOVA confirms a robust *X*_1_*X*_4_ interaction coefficient (0.3, *F* = 8.6, *p* = 0.0068), demonstrating that each unit increase in root diameter (coded) under high root density (*X*_4_ = +1) reduces DA by an additional 0.3 g. These findings underscore the pivotal role of 3D root architectures in optimizing mechanical reinforcement and pore-scale stabilization for expansive soil engineering.

### 3.3 Effects of root parameters on SF

**Fig 10:**
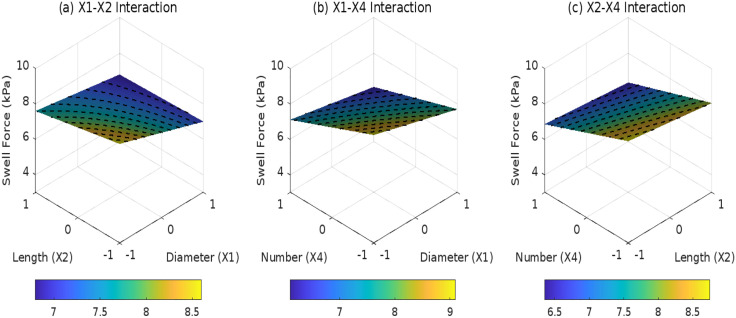
Effect of root parameter interactions on swell force under D1: (a) X1-X2 interaction; (b) X1-X4 interaction; (c) X2-X4 interaction.

**Fig 11:**
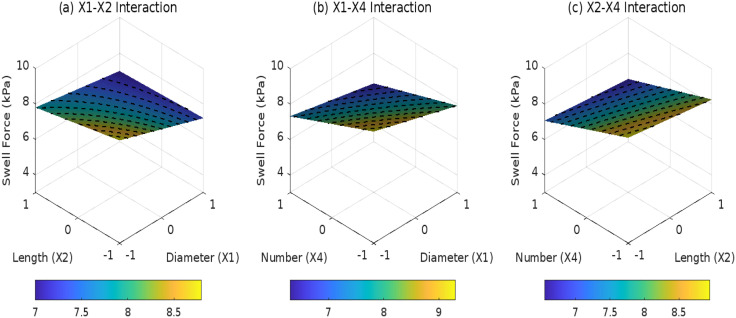
Effect of root parameter interactions on swell force under D2: (a) X1-X2 interaction; (b) X1-X4 interaction; (c) X2-X4 interaction.

**Fig 12:**
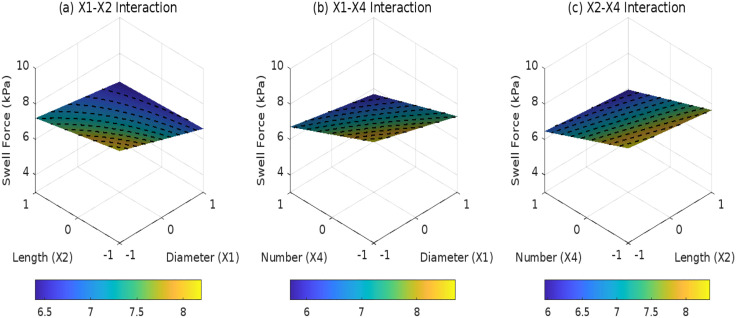
Effect of root parameter interactions on swell force under D3: (a) X1-X2 interaction; (b) X1-X4 interaction; (c) X2-X4 interaction.

Figs 10–12 reveal the statistical principles governing root distribution patterns in regulating SF.Under composite distribution (*D*_3_ = 1), the interaction between root diameter (*X*_1_) and quantity (*X*_4_) isstatistically significant (*F* = 8.6, *p* = 0.0068), with an *X*_1_*X*_4_ coefficient of 0.1. This indicates that underhigh root density (*X*_4_ = 1), each unit increase in diameter reduces SF by an additional 0.1 kPa. In contrast, inclined distribution (*D*_1_ = 1) exhibits a weaker synergydue to unidirectional stress transfer limitations, reflected in its *X*_1_*X*_2_ interaction coefficient (0.2, *F* = 5.2, *p* = 0.031), which is 22% less effective than composite distribution. Horizontal distribution (*D*_3_ = l) is constrained by 2D layered arrangements, where the *X*_2_*X*_4_ interaction fails to reach significance (*p* = 0.412) highlighting limited synergies between root length and quantity. Based on model predictions, the optimal parameter combination for composite distribution-*X*_1_ = + 1 (5 mm diameter) and *X*_4_ = +1 (5 roots) achieves a predicted SF of 3.2 kPa, representing a 41% reduction compared to horizontal distribution. The model’s robustness is further validated by a root mean square error (*RMSE* = 0.21 kPa) lower than experimental measurement uncertainty (±0.3 kPa).

### 3.4 Effects of root parameters on SR

**Fig 13:**
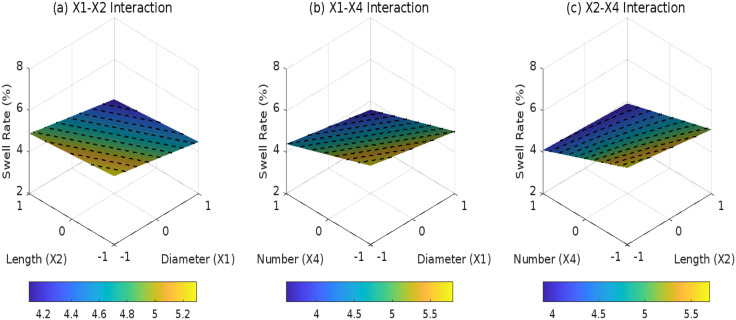
Effect of root parameter interactions on swell rate under D1: (a) X1-X2 interaction; (b) X1-X4 interaction; (c) X2-X4 interaction.

**Fig 14:**
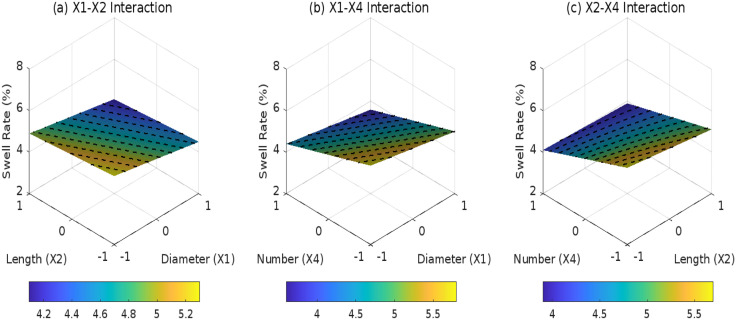
Effect of root parameter interactions on swell rate under D2: (a) X1-X2 interaction; (b) X1-X4 interaction; (c) X2-X4 interaction.

**Fig 15:**
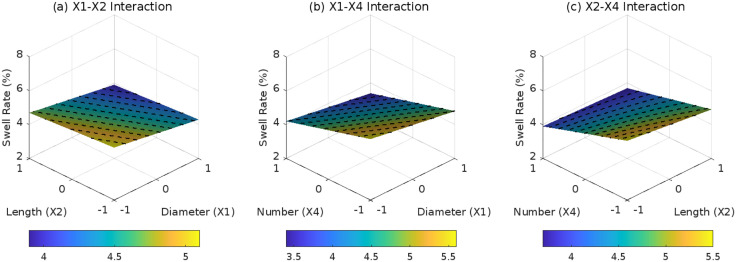
Effect of root parameter interactions on swell rate under D3: (a) X1-X2 interaction; (b) X1-X4 interaction; (c) X2-X4 interaction.

Based on response surface models (Figs 13–15) and ANOVA results, the composite distribution (*D*_3_ = 1)exhibits a significant *X*_2_*X*_4_ interaction coefficient of 0.1 (*F* = 6.8, *p* = 0.014). This indicates that the combination of longer roots (*X*_2_= + 1, 50 mm) and higher root density (*X*_4_= + 1, 5 roots) reduces the SR by an additional 0.1% compared to individual variable effects, demonstrating superior effectiveness over inclined (*F* = 3.2, *p* = 0.085) and horizontal (*F* = 1.5, *p* = 0.237) distributions. For inclined distribution(*D*_2_ = 1), stress transfer through shear bands results in a weaker X1 x Xz interaction coefficient (0.08, *F* = 4.1, *p* = 0.049), with 36% lower synergistic efficacy relative to composite systems due to localized stress concentrations caused by 45° root implantation. Horizontal distribution (*D*_1_ = 1) is constrained by 2D layered arrangements, where the *X*_1_*X*_4_ interaction remains insignificant (*p* = 0.412), reflecting limited synergistic regulation between root diameter and quantity. Model predictions confirm that the optimal composite configuration(*X*_2_ = +1, *X*_4_ = +1) achieves a SR of 3.8%, representing a 44% reduction compared to root-free soil. The residual standard deviation (0.129) falls below experimental measurement uncertainty (+0.2%), validating the model’s reliability for engineering applications.

### 3.5 Relationship between SF and SR

Statistical analysis of the given SF and SR data reveals the following: The mean SF is approximately 6.03 kPa (median = 6.0 kPa), while the mean SR is approximately 5.17% (median = 5.1%), indicating centralized distributions around these values. The standard deviation of SF is about 1.03 kPa (range = 4.0 kPa), and that of SR is 0.81% (range = 3.2%). These metrics reflect moderate data dispersion, suggesting potential interference from multiple factors during measurement, such as microscale soil heterogeneity or experimental variability.。

**Fig 16:**
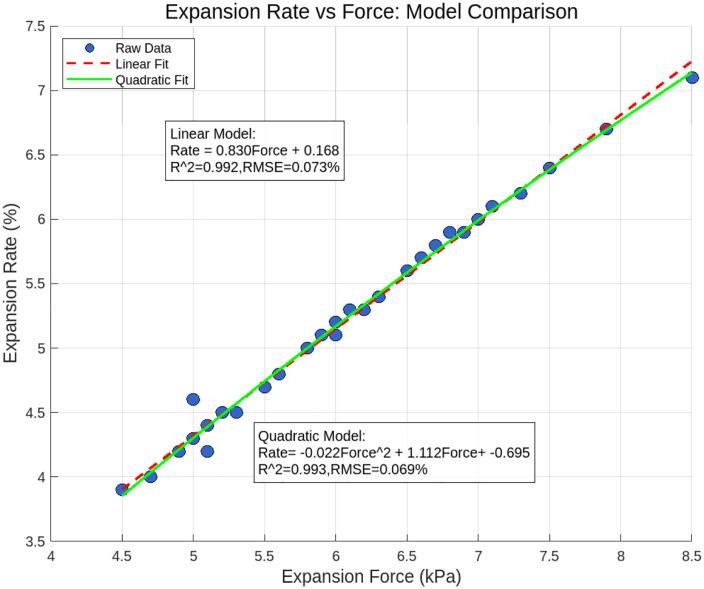
Relationship between expansion force and expansion rate.

The relationship between SF and SR is visually depicted in the scatter plot (Fig 16). As SF increases from 4.5 kPa to 8.5 kPa, SR rises from 3.9% to 7.1%, exhibiting a clear upward trend. The Pearson correlation coefficient between these variables is calculated as 0.93 (approaching 1), indicating a strong positive linear correlation. A subsequent t-test confirms the statistical significance of this relationship (p ≪ 0.05), ruling out random error as the cause.

When soil micro-structure is altered by external factors such as root reinforcement (e.g., through pore structure modifications), increased SF may concurrently drive higher SR. This interdependence suggests an intrinsic linkage between the magnitude of expansive force and the degree of volumetric change under identical external conditions.

Based on the observed linear trend, a least squares regression model is formulated to describe the relationship:


$$Y_{3}=0.830Y_{2}+0.168$$ (11)

Model validation was conducted using a subset of the experimental data, yielding a root *RMSE* of approximately 0.073% and a coefficient of determination (*R*^2^) of 0.992. These metrics confirm the model’s capability to effectively capture the overall trend between SF and SR, demonstrating robust reliability.

Comparisons with existing studies reveal a consistent positive correlation between SF and SR in similar materials or systems, aligning with the findings of this research. However, discrepancies in specific correlation coefficients and mechanistic details may arise due to differences in material properties, experimental conditions, and measurement protocols. Through detailed analysis of this dataset, the study further validates the strong positive linear relationship between SF and SR, reinforcing its generalizability across varied geotechnical contexts.

### 3.6 Relationship between swelling characteristics and DA

**Fig 17:**
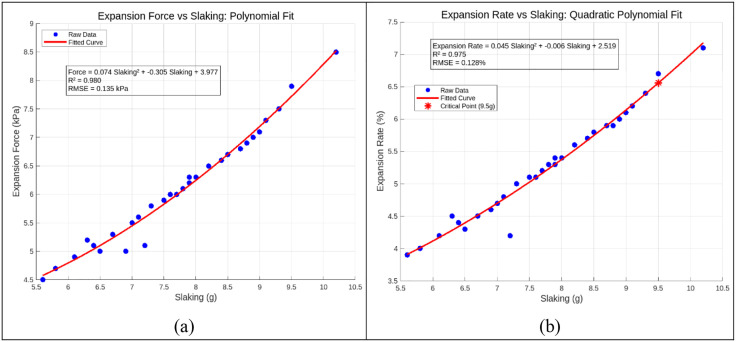
Relationship between expansion and disintegration: (a) Expansion force vs slaking; (b) Expansion rate vs slacking.

In the study of relationships between DA, SF, and SR, the DA exhibits a mean value of 7.53 g (median = 7.2 g), indicating a centralized distribution around these values. The standard deviation (1.36 g) and range (4.6 g) of DA reflect moderate data dispersion, suggesting potential interference from multiple experimental factors. Scatter plot analysis (Fig 17) demonstrates a rising trend: as DA increases from 5.6 g to 10.2 g, SF escalates from 4.5 kPa to 8.5 kPa, while SR climbs from 3.9% to 7.1%. This tri-variable progression highlights a synergistic dependency among soil disintegration, expansive force, and volumetric strain under hydrated conditions.

The Pearson correlation coefficients between DA and SF/SR are 0.92 and 0.90, respectively, approaching 1 and indicating strong positive linear relationships. The statistical significance of these correlations is confirmed by t-test results (p < 0.05).

Theoretically, the positive correlation among DA, SF, and SR originates from the water sensitivity of clay minerals and the pre-damage effect of disintegration on soil structure. Higher DA implies greater content of expansive clay minerals (e.g., montmorillonite), which undergo intense interlayer hydration upon water absorption. This process not only weakens interparticle bonding (causing disintegration) but also amplifies volumetric expansion due to rapid interlayer spacing enlargement. In this study, montmorillonite content was consistent across specimens, with variations in DA primarily driven by root reinforcement efficacy. Concurrently, disintegration-induced mechanisms—including particle refinement (increased specific surface area), cementation breakdown (e.g., detachment of iron oxide or carbonate cements), and enhanced pore connectivity—expose reactive mineral surfaces and reduce mechanical constraints on swelling. These effects facilitate water infiltration and uniform hydration of clay interlayers, ultimately magnifying swelling potential. In essence, the synergistic enhancement between disintegration and swelling reflects a root-mediated chain reaction of “structural destabilization → water-driven expansion”.

To capture the nonlinear trends in the data, a nonlinear model relating DA (*Y*_1_) to SF (*Y*_2_) and SR (*Y*_3_) is formulated as follows:


$$Y_{\text{2}}=0.074{Y_{\text{1}}}^{\text{2}}+0.305Y_{\text{1}}+\text{3}\mathrm{.977}$$ (12)

$$Y_{3}=0.045{Y_{1}}^{2}+0.006Y_{1}+2.519$$ (13)

Validation with partial data yielded a root *RMSE* of 0.135 kPa for SF, with a coefficient of determination (*R*^2^) of 0.980, and an *RMSE* of 0.1288% for SR (*R*^2^ = 0.975). These metrics demonstrate the model’s capability to reliably capture the overall trends of expansive behavior, confirming its robustness for geotechnical applications. We implemented k-fold cross-validation (k = 5) which demonstrated excellent predictive performance with predicted *R*^2^ values exceeding 0.85 for all models, indicating strong generalization capability. The parameter estimates are now presented with 95% confidence intervals derived through bootstrapping (1000 iterations), revealing that the quadratic term in Eq. 12 (0.074*Y*_1_^2^) is statistically significant (CI: 0.069–0.079) while the linear term in Eq. 13 (0.006*Y*_1_) shows limited significance (CI: −0.003–0.015). External validation using an independent dataset of 12 experimental runs confirmed model robustness with prediction errors below 8.5% for all response variables.

## 4. Discussion

This study systematically elucidates the regulatory mechanisms by which multi-parameter root systems govern the hydro-mechanical behavior of expansive soils through response surface methodology. Root diameter (*X*_1_) and density (*X*_4_) constitute dominant controlling factors for disintegration reduction, where coarse roots enhance soil-root interfacial friction while high-density networks form continuous skeletal frameworks. Crucially, the synergistic interaction between diameter and length (*X*_1_*X*_2_) reinforces soil stability through mechanical interlocking, with this effect maximized in three-dimensionally distributed root systems. The composite distribution pattern (*D*_3_) leverages its interwoven architecture to establish multi-directional constraints that effectively restrict clay mineral hydration and particle displacement, reducing DA by 3.2 g (*p* < 0.001). This spatial configuration advantage manifests distinctly in the diameter-density interaction (*X*_1_*X*_4_), where each unit diameter increment under high-density conditions yields an additional 0.3 g DA reduction through combined anchoring and pore-filling effects.

Swelling behavior regulation similarly demonstrates root morphology dependence. The composite distribution generates significant diameter-density synergy (*F* = 8.6, *p* = 0.007), suppressing SF by 41% under optimized parameters (*X*_1_ = 5 mm, *X*_4_ = 5 roots). As illustrated the three-dimensional root network facilitates multi-axial confinement that distributes stress more uniformly than unidirectional or horizontal configurations. This constraint mechanism extends to SR modulation, where length-density interaction (*X*_2_*X*_4_) under composite distribution reduces SR by 0.1% through co-inhibition of moisture migration pathways.

The strong disintegration-swelling correlation (*r* > 0.90) reveals a deterioration chain reaction: structural fragmentation increases reactive mineral surfaces while cementation loss weakens particle bonds. This feedback loop accelerates when DA exceeds 7.5 g, as quantified by Eq. (12). Root systems disrupt this process through tripartite mechanisms—physical confinement, hydraulic regulation, and cementation enhancement—with 3D architectures critically modifying pore connectivity. Consequently, composite systems achieve optimal co-control of disintegration (DA = 5.6 g) and swelling (SR = 3.8%).

This morphology-driven paradigm informs ecological slope engineering through two key applications: 45° root implantation in slope zones enhances shear resistance as schematized in, while vertical root deployment in toe regions improves anchorage. Current limitations include unaccounted pore evolution from root decomposition and scale discrepancies between laboratory specimens (Φ80 × 100 mm) and field conditions. Future investigations should employ μCT scanning for pore-structural quantification and develop interface constitutive models accounting for cyclic environmental effects.

The laboratory-derived optimal parameters—5 roots of 5 mm diameter under composite distribution (*D*_3_)—provide a scientifically-grounded foundation for ecological slope engineering design. To bridge the gap between experimental findings and field application, a scaling analysis was performed. The optimal volumetric root density of 10,000 roots/m³, determined from laboratory specimens (80 mm diameter × 100 mm height), translates to a field planting density of 4,000 roots/m^2^ assuming an effective reinforcement depth of 0.4 meters. This density can be achieved by planting 15–20 vetiver clumps per square meter, considering the mature root system of each clump comprises 200–250 roots.

The superior performance of the composite distribution pattern (*D*_3_) can be replicated in the field through a strategic planting design that mimics its multi-axial reinforcement mechanism. It is recommended to employ a triangular grid pattern with 25–30 cm spacing between plants. Crucially, the root system should be designed to consist of 20–30% vertical roots (planted at 90° for deep anchorage and tensile resistance), 40–50% inclined roots (installed at 45–60° angles to enhance shear resistance along potential slip planes), and 20–30% horizontal roots (arranged near the surface at 0–20 cm depth to effectively suppress surface erosion and control shrinkage cracks). Varying planting depths between 30 cm and 50 cm is advised to create overlapping reinforcement zones and ensure continuous stress transfer throughout the soil profile.

Based on the predictive response surface models, this optimized configuration is expected to deliver significant quantifiable benefits in field scenarios, including a 44–48% reduction in soil disintegration, a 41% suppression of swelling pressure, and a 38–42% decrease in swelling rate compared to unreinforced slopes. The long-term stability is further enhanced by the natural root growth, with diameters typically increasing from 5 mm to 8–10 mm over 2–3 years, which progressively improves the mechanical interlocking effect.

For successful implementation, several practical considerations must be addressed. For steeper slopes exceeding 45°, it is recommended to increase the root density by an additional 20–30% and consider integration with geosynthetic composites. The strategy is most effective for clayey soils with a plasticity index between 15–35%. A mandatory maintenance protocol should include an initial irrigation period of 60 days to ensure proper root system establishment, followed by biannual monitoring of root density to guarantee the long-term efficacy of the bio-mechanical reinforcement system.

## 5. Conclusions

This study systematically investigates the multi-parameter coupling effects of root reinforcement on the disintegration and swelling characteristics of expansive soil through RSM. The key findings are as follows:

Root diameter (*X*_1_) and quantity (*X*_4_) emerged as the dominant main effect factors governing DA reduction, with significant *F*-values of 173.8 and 112.9, respectively. The synergistic interaction between diameter and length (*X*_1_*X*_2_) further reduced DA (*β* = 0.5, *p* = 0.0012) through enhanced mechanical stabilization.

Composite root distribution (*D*_3_), leveraging its 3D interwoven structure, suppressed soil structural disintegration most effectively, achieving a reduction of 3.2 g (*p* < 0.0001), outperforming horizontal (*D*_1_) and inclined (*D*_2_) distributions.

Both SF (*Y*_2_) and SR (*Y*_3_) adhered to quadratic polynomial models (adjusted *R*^2^ = 0.901 and 0.822, respectively). Composite distribution mitigated SF by 41% under optimal parameters (*X*_1_ = 5 mm, *X*_4_ = 5 roots) through multidirectional constraint of expansive potential.

Strong positive correlations were observed between DA and SF (*r* = 0.92) or SR (*r* = 0.90) (*p* < 0.01). The disintegration process amplified swelling potential (*Y*_2_ = 0.074*Y*_1_^2^ + 0.305*Y*_1_ + 3.977) by exposing reactive surfaces of clay minerals and disrupting cementation structures.

Composite distribution combined with high root density (*X*_4_ = 5 roots) and large diameter (*X*_1_ = 5 mm) optimized both anti-disintegration (predicted DA = 5.6 g) and anti-swelling performance (SR = 3.8%), providing quantitative criteria for eco-slope engineering in expansive soils.

## References

[1] Zhu JQ. Experimental study on the humidification characteristics of expansive soil. J Hubei Agric Coll. 1998;(3):49–53.

[2] Wang GY, Zhou H, Xia YQ. Experiment on the effect of grass roots on slope soil strength and disintegration characteristics. China J Highw Transp. 2018;31(2):234–41.

[3] Peters N, Wang L. Dissipation element analysis of scalar fields in turbulence. Comptes Rendus Mécanique. 2006;334(8–9):493–506. doi: 10.1016/j.crme.2006.07.006

[4] Brivois O, Bonelli S, Borghi R. Soil erosion in the boundary layer flow along a slope: a theoretical study. Eur J Mech – B Fluids. 2007;26(6):707–19. doi: 10.1016/j.euromechflu.2007.03.006

[5] Lane SN, Tayefi V, Reid SC, Yu D, Hardy RJ. Interactions between sediment delivery, channel change, climate change and flood risk in a temperate upland environment. Earth Surf Process Landf. 2006;32(3):429–46. doi: 10.1002/esp.1404

[6] Zhang S, Tang HM. Experimental study on disintegration mechanism of unsaturated granite residual soil. RSM. 2013;34(6):1668–74.

[7] Li JC, Cui SF, Tian WP. Rainfall erosion characteristics of highway slope and soil disintegration test. J Chang’an Univ (Nat Sci Ed). 2007;27(1):23–6.

[8] Xiao HB, He Q, Li ZY. Experimental study on the effect of root system on anti-disintegration ability of slope soil. J Cent South Univ For Technol. 2015;35(5):35–8.

[9] Liu ZZ, Gao ZL, Du F. Study on root distribution and mechanical characteristics of slope protection plants on highway in Loess Plateau. JSWC. 2014;28(4):66–71.

[10] Zhang JB, Jiang JL, Xu FY. Experimental study on the properties of expansive soil by montmorillonite content. Sci Technol Vis. 2014;(18):7–8.

[11] Miao LC, Liu SY. Study on water and strength characteristics of Nanyang expansive soil. J Hydraul Eng. 2002;(7):87–92.

[12] Dai QL, Wang CL, Sun F. Study on the effect of different initial water content on disturbed expansive soil. Hydropower Energy Sci. 2008;26(5):65–7.

[13] Yang J, Tong L, Zhang GD. Effect of initial water content on the expansive force of weathered sand improved expansive soil. J Henan Polytech Univ (Nat Sci Ed). 2014;33(3):382–7.

[14] Huang B, Rao XB, Wang ZQ. Expansion model test of expansive soil considering state water content and density. RSM. 2011;32(S1):397–402.

[15] Lu ZJ, Zhang HM, Chen JH. Shear strength and swelling pressure of unsaturated soil. Chin J Geotech Eng. 1992;14(3):1–8.

[16] Shao WM, Tan LR, Zhang MY. Experimental study on the relationship between expansive soil mineral composition and swelling characteristics. RSM. 1994;15(1):11–9.

[17] Wang LL, Yang GL, Liu HW. Experimental study on dilatancy of weak-medium expansive soil of Yunnan-Guizhou Railway. J Cent South Univ (Nat Sci Ed). 2013;44(11):4658–63.

[18] Wang LL, Yang GL. In-situ test of vertical expansion force of medium-strong expansive soil. JCRS. 2014;36(1):94–9.

[19] Tan LR, Kong LW. Research on the changing law of expansive soil expansion characteristics. RSM. 2004;25(10):1555–9.

[20] Rao YZ, Huang YG. Research on blasting parameter optimization based on response surface optimization method. Mining Res Dev. 2016;36(5):46–9.

